# Psychological well-being of travellers on long-distance shipping voyages: A systematic review protocol

**DOI:** 10.12688/f1000research.153730.1

**Published:** 2024-10-07

**Authors:** Audifax Kpeno, Twinkle Rout, Pratap Kumar Sahu, Nachieketa Khamari Sharma, Surjeet Sahoo, Deepak Kumar Pattanaik

**Affiliations:** 1 Psychiatry, Siksha O Anusandhan University, Bhubaneswar, Odisha, India; 2 Pharmacy, Siksha O Anusandhan University, Bhubaneswar, Odisha, India; 3 Pharmacy, Siksha O Anusandhan University, Bhubaneswar, Odisha, India; 4 Physics, Siksha O Anusandhan University, Bhubaneswar, Odisha, India

**Keywords:** Psychosocial, well-being, mental-health, shipping voyages, Seafarers

## Abstract

The psychosocial well-being of every individual is as important as the well-being of long-distance voyages, especially that they have to spend longer hours and days travelling to their destinations with all the experiences of adventure and adversities characterizing the journey. The aim of this systematic review is to explore the psychosocial well-being of long-distance travellers on board shipping vessels to gain an understanding of their psychological and social well-being with the objective of ameliorating the adversities associated with these travels. A systematic review will be conducted following the Preferred Reporting Items for Systematic Reviews and Meta-Analyses (2020) statement. The databases to be searched for the data will be limited to Scopus, Web of Science, and Advanced Google Scholar using keywords selected by the reviewers. Meta-analysis, narrative synthesis, or both will be used depending on the extent of heterogeneity across eligible observational studies included in the review. Some specific countries will be selected for data extraction, and only data published in English will be included. It is expected that the findings from this review will bring to bear if these categories of travellers are prone to risks that affect their psychosocial wellbeing and if so discovered.

This protocol was registered with the PROSPERO. **PROSPERO registration number: CRD42024517277(08/03/2024)**

**Abbreviations**
HAM-A: Hamilton Anxiety Rating Scale
HAM-D: Hamilton Depression Rating Scale
JDI: Job Descriptive Index
JIG: Job In General
MSQ: Minnesota Satisfaction Questionnaire
PHQ: Patient Health Questionnaire
PRISMA: Preferred Reporting Items for Systematic Reviews and Meta-Analyses
WHO: World Health Organisation

## Introduction

The role of seafarers is peculiar in that their work is not restricted by time; they are constantly working both onshore and offshore. Hence, they find their social lives circumscribed, ^1^ predisposing them to a myriad of psychological and physical challenges. ^2^ Seafarers are often onboard a ship for several long hours faced with adverse weather conditions such as “high pitched noises, vibrations, cold spells, high temperatures and unstable moisture conditions” ^3^ which does not augur their physical health.

Settling on a definition for long distance travel has been described as challenging ^4^ in a white paper where long-distance travel has been defined broadly as “out-of-town” trips. These trips have been limited to intercity or inter-regional trips and may not involve overnight stays. ^4^ However, for the purpose of our review, long-distance voyages are defined to include maritime journeys that involve the transportation of goods of people across vast stretches of the ocean, typically spanning significant distances between distant ports or destinations. In addition to this definition, these voyages often entail extended durations at sea and may traverse multiple continents or regions.

Sea travel or travel by ocean on long-distance shipping voyages is a journey characterized by both adventure and adversity. Notwithstanding all the turbulence associated with sea travel, the sea is seen as a temporary home for those who have to undertake long-distance travel. An exploration of the psychological well-being of those who call the sea their temporary home cannot be overly emphasized, as it will help bring out the complexities associated with shipping voyages. This will shed light on the various dimensions that shape their mental and emotional states amidst the challenges of maritime life.

At the crust of this journey of exploration lies the concept of well-being, defined as a multidimensional construct that serves as a compass guiding individuals towards a life of satisfaction, happiness, and overall quality. ^5^ Rooted in both subjective experiences and objective indicators, well-being includes both internal and external factors that shape one’s perception of life satisfaction and fulfilment. ^5^ As travellers embark on their maritime journeys, their well-being is linked to their ability to overcome the challenges of life at sea while maintaining a sense of inner equilibrium and contentment.

Complementing the exploration of well-being is the examination of the concept of psychosocial well-being, which encompasses the mental, emotional, and social dimensions of health. Embedded within this construct are psychological factors such as self-esteem and coping mechanisms, social factors such as social support and interpersonal relationships, and emotional factors such as mood regulation and stress management. ^6^ As travellers face the challenges of long-distance shipping voyages, their psychosocial well-being serves as a guide, providing support and direction to cope with the challenges and changes that come with living and working on a ship, influencing their ability to adapt, connect, and thrive amid the vast expanse of the open sea.

### Objective

This review seeks to achieve the following objectives: to assess the prevalence of psychosocial issues among travellers on long-distance shipping voyages; to identify the factors contributing to psychosocial challenges among travellers; and to examine the impact of psychosocial issues on the well-being and performance of travellers to evaluate existing interventions and support systems for promoting psychosocial well-being among travellers. We intended to answer the following research questions:

Q1: What are the subjective experiences of seafarers during long-distance voyages and how do they perceive and cope with the challenges they encounter?

Q2: What role do social support networks play in mitigating psychosocial challenges among seafarers and how do these networks operate within the maritime environment?

Q3: What are the key factors influencing psychosocial well-being among seafarers, including work-related stressors, voyage duration, and interpersonal relationships?

## Methods

This systematic review will be conducted considering the standard reporting format of the preferred reporting items for systematic review and meta-analysis (PRISMA, 2020) protocol guidelines.

### Eligibility criteria

We established specific eligibility criteria to inform the inclusion of studies in the review. Our criteria were designed to ensure that the included studies focused on individuals engaged in long-distance shipping voyages and investigated psychosocial factors, experiences, or interventions related to this context. Studies that capture individuals, such as seafarers, crew members, and maritime workers employed in long-distance shipping voyages, are included. For example, studies exploring the impact of isolation, confinement, cultural adaptation, job stress, social support, mental health services, and coping mechanisms among individuals on long-distance shipping voyages were considered eligible.

On the other hand, we will exclude studies that focus solely on populations not directly related to long-distance shipping voyages, such as studies on short-distance maritime workers or land-based occupations. Additionally, studies focusing solely on passengers or tourists rather than crew members or maritime workers were excluded from our review.

Furthermore, to ensure that the selected studies align with the objectives of our review, we included studies that measured various aspects of psychosocial well-being, including but not limited to mental health, emotional well-being, job satisfaction, social connectedness, quality of life, stress, burnout, resilience, and coping strategies among individuals engaged in long-distance shipping voyages. This encompasses the exploration or measurement of factors such as depression, stress, anxiety, social support, and coping mechanisms, aligning with the objectives of this review. Additionally, eligible studies should measure the prevalence of psychosocial well-being among shipping voyages and address the aspects of job satisfaction and quality of life related to long-distance voyages. Studies lacking relevant psychosocial well-being outcomes or measures, as well as those focusing solely on organizational factors, technical aspects, or non-psychosocial aspects of maritime work, were excluded from our review.

### Definition of outcomes

The outcomes of this review will include measures of depression, anxiety, social support, job satisfaction, and QoL. Studies that provide acceptable standard definitions for these concepts were considered. Definitions for depression and anxiety should not deviate widely from those given by Beck, ^7^ as this definition is considered the most acceptable. Quality of life should be defined by the World Health Organization (WHO). It should encompass an individual’s subjective life experiences, including their physical, mental, social, and emotional well-being. ^8^ Studies that have operationalized these constructs with standardized scales, including but not limited to the HAM-A and HAM-D for anxiety and depression, respectively, the Patient Health Questionnaire (PHQ), ^9^ Job Descriptive Index (JDI), ^10^ Job In General (JIG) scale, ^11^ and Minnesota Satisfaction Questionnaire (MSQ) ^12^ will all be included in this review.

### Types of study

Primary studies that used qualitative or quantitative methods will be included in the review. This allowed a broad range of studies to be included. Mixed-methods studies are also eligible for inclusion, as they can provide valuable insights by combining qualitative and quantitative approaches.

### Participants

This review will include all studies that used participants who have had long-distance shipping voyages, regardless of gender or geographical orientation. However, it will exclude children below the age of 15, as they may not be able to corroborate their experience well enough to inform generalization.

### Study setting

This review includes studies conducted in India, Denmark, the United Kingdom, Sweden, Ireland, Germany, South Africa, Norway, and the Czech Republic. These countries were selected because of their high shipping voyage rate. Particular attention of the reviewers is paid to India because we intend to leverage the results of this review to conduct primary research within the Indian shipping voyage space.

### Sources of data

The researchers intended to develop search strategies for eligible literature using keywords related to maritime activities and mental health. Databases, such as Scopus and Web of Science, will be searched using keywords. These databases were selected as they are considered to hold large volumes of data worldwide, so the potential of covering most of the studies is very high. Advanced Google search engines will also be searched to capture other data that otherwise might not have been indexed in these two major databases (Scopus and Web of Science). Dissertations and theses were excluded because the researchers intended to work with only published records.

This systematic review focuses exclusively on published records and peer-reviewed journal articles. Unpublished theses and dissertations will be excluded from the review owing to potential limitations in access, quality control, or potential bias in unpublished work. All searches will be limited to studies published in English and will involve only human subjects. It is anticipated that this review will be completed in September 2024. That is, the expected end-year search for the data will end in 2024.

### Search strategy and process

The literature search will employ Boolean Operators (AND and OR) along with side keywords for each of the databases to be searched. For instance, psychosocial OR “well-being” OR “mental-health” OR “Shipping Voyages” OR Seafarers; for the first phase of the search; the other phase will combine keywords such as psychological AND “shipping voyages” AND Seafarers. This formula was applied to both databases from which the search was carried out. The search was limited to countries in the study setting.

The search was conducted in two phases. The initial phase will focus on identifying studies conducted within India, given the specific context and relevance of the topic to this geographic region, as well as the fact that the researcher intends to rely on the findings of this review to carry out an original study. Subsequently, a broader search will be carried out to capture relevant literature from other named countries, as indicated in the study setting, to ensure comprehensive coverage of the available evidence.

The statement for Reporting Literature Searches in Systematic Reviews (PRISMA-S) ^13^ will be relied upon as a guide for reporting our search results. The two authors (AK and TR) will carry out the search independently, and AK and PD will compile the search results and remove duplicates.

### Data selection process

At this stage of the review, three authors, AK, TR, and PD, will independently screen the titles and abstracts of the identified records using eligibility criteria. Subsequently, they will independently screen the full text of other records for which decisions could not be reached after their titles and abstracts were screened. On one hand, through a one-on-one interaction between PD, AK and TR, a discussion will be held to resolve any discrepancies that would arise during the screening process. After reaching an agreement on the records to be included by these three authors, NKS and SS will give a final validation to these records owing to their expertise in maritime and Psychological research respectively.

### Risk of bias assessment in studies extracted

To ensure that the findings of all the records extracted are free from the risk of bias, based on reliable and unbiased evidence, two of the reviewers, NKS and PKS, will use the Cochrane Collaborations’ Risk of Bias tool ^14^ to systematically evaluate the risk of bias in the extracted records. This must be performed to meet the standard set for the systematic review protocol. In a systematic review, minimizing bias is a core component of the review, ^15^ and one of the most preferred means to achieve this is by employing the most appropriate tools. Many tools exist for the execution of this process. However, the Cochrane Collaboration Risk of Bias tool is the most preferred and widely used tool. This affords researchers the opportunity to consider plausible limitations of the studies to be included in the review in order to obtain reliable conclusions.

### Quality assessment

To determine the reliability and credibility of the individual documents, a quality assessment was performed by two authors with several years of experience in the field of psychiatry. One performed the initial assessment, and the other crosschecked. The quality rating was premised on the following criteria: “Selection bias,” “random sequence generation,” “allocation concealment,” “reporting bias,” “other bias,” “performance bias,” “detection bias,” “data collection methods” and “attrition bias”. ^11^ Each of the articles included in the study was assessed for bias using the Cochrane Collaboration Risk of Bias tool.

### Data synthesis

Our choice of approach for data synthesis is informed by the degree of heterogeneity observed. We will use meta-analysis, narrative synthesis, or both. Meta-analysis, a statistical method employed when combining results of large samples of data (records) to be included in a study, provides, to a larger extent, the reliability or precision of the estimates on any treatment effects. ^16^ Meta-analysis involves pooling data from individual studies, analyzing them collectively, and often calculating a weighted average effect size. ^16^ On the other hand, narrative synthesis involves a qualitative approach to synthesize evidence from multiple studies. Here, rather than pooling data and collecting statistical analyses, narrative synthesis focuses on summarizing and interpreting findings from individual studies in a narrative format. It includes the description of patterns, themes, and discrepancies across studies, providing a qualitative understanding of the topic under investigation. ^16^

The researchers’ choice of any or both of these methods is contingent on the outcome of the selected records. However, in the event where heterogeneity will be observed among studies, it will be incumbent to use narrative synthesis, since the application of meta-analysis in this situation will be challenging as the studies will be too different to be combined. In the same instance of heterogeneity prevalence, the researcher will follow the three-step approach to narrative synthesis to synthesize the results included in the study. ^17^ These steps include organizing the description of the studies into logical categories, analyzing the findings within each of the categories, and synthesizing the findings across all included studies. ^17^ Tables and graphs are used to support narrative synthesis or qualitative summary.

## Discussion

This discussion will focus on three thematic areas: beginning with the implications for this planned review, the significance of the topic, and addressing the research objectives of the review.

This systematic review has significant implications for research, practice, and policies in several areas. From a research perspective, the findings of this review have the potential to advance knowledge in maritime health and occupational psychology. Identifying factors contributing to psychosocial challenges among travellers can guide future research efforts to explore causal mechanisms, risk factors, and protective factors associated with well-being in this population. Additionally, the review highlights gaps in the literature and opens further investigation into the understudied aspects of psychosocial well-being among travellers.

In terms of practice, the review findings can inform the development of interventions and support strategies aimed at promoting the well-being of travellers on long-distance shipping voyages. This review will delve into synthesizing effective practices and identifying areas for improvement to guide shipping companies, healthcare providers, and other stakeholders in their implementation of effective strategies to address psychosocial challenges such as isolation, stress, and cultural differences. From a policy perspective, this review provides valuable insights into the regulatory frameworks and guidelines needed to ensure the health and well-being of travellers in the maritime industry.

This review will shed light on the potential impact of psychosocial issues on the health, safety, and performance of individuals in this population, as well as the broader implications for maritime industry stakeholders, regulatory agencies, and policymakers. Applied psychology research has extensively explored stress-related factors within transportation contexts, including those related to drivers and commuters. While much of this research has centered on terrestrial transportation, there are parallels to be drawn with voyages on long-distance shipping. The maritime environment, characterized by prolonged periods away from home, isolation, and the demands of shipboard life, presents unique psychosocial challenges for individuals. Understanding the impact of these stressors on voyagers’ well-being and performance is crucial for ensuring the safety and efficiency of maritime operations. Psychological factors, such as personality disposition, attitudes towards voyage destinations, and coping mechanisms, play significant roles in shaping the experiences of travellers. Moreover, issues such as fatigue, boredom, and loss of alertness can have profound effects on the operational effectiveness and safety at sea. By addressing these psychosocial issues, this review aims to provide insights that will guide maritime industry stakeholders in making informed policy decisions to support voyagers’ well-being and enhance operational outcomes.

This review systematically examines the prevalence of psychosocial issues among travellers on long-distance shipping voyages by reviewing existing evidence from relevant studies to provide insights into the frequency and scope of these issues within the target population. Through a thorough examination of the literature, this review identifies the factors that contribute to psychosocial challenges among travellers. We will synthesize findings from diverse studies, including qualitative research, observational studies, and quantitative analyses, to gain an appreciable understanding of the contributing factors, such as isolation, confined living spaces, job stress, and cultural differences. This review will assess the impact of psychosocial issues on the well-being and performance of travellers by synthesizing evidence on relevant outcomes, such as mental health, emotional well-being, job satisfaction, and job performance. As we analyzed the reported associations between psychosocial challenges and these outcomes, this review will provide insights into the broader implications for individual health, safety, and organizational effectiveness.

The PRISMA-P Checklist for the systematic review protocol ^18^ has been attached as an extended data. This protocol was registered with the PROSPERO. Prospero registration number: CRD42024517277

## Disclosure

All authors have approved the final article.

## Ethics and consent

Ethical approval and consent were not required

## Strengths and limitations of the review

• The review will be reported in accordance with the PRISMA frame protocol
• This review will add to the few reviews, if not the first of its kind, regarding the psychosocial well-being of long-distance travellers.
• This review will examine three senior experienced researchers contributing to the data extraction and inclusion process to ensure that the evidence that will be adduced will stand the test of time.
• The exclusion of some of the databases in the data extraction process may hinder the inclusion of records that might not have been indexed in the three databases employed.

## Disclosure

All authors have approved the final article.

## Availability of data

This is a protocol, and there were no data at this stage of the study.

## Data Availability

No data are associated with this article. ZENODO: Prisma – P Checklist for Psychological well-being of travelers on long-distance shipping voyages: A systematic review protocol, https://doi.org/10.5281/zenodo.13838158 ^18^ Data are available under the terms of the Creative Commons Attribution 4.0 International license (CC-BY 4.0).
