# Supplementary material for: Psychological well-being of travellers on long-distance shipping voyages: A systematic review protocol
Source: F1000Res. 2024 Nov 8;13:1147. Originally published 2024 Oct 7. [Version 2] doi: 10.12688/f1000research.153730.2 (PMC11574325; doi:10.12688/f1000research.153730.2)
Supplement: Peer review history

# Reviewer report: Reviewer response for version 2

**DOI:** 10.5256/f1000research.174047.r338885

**Published:** 2024-11-18

**Authors:** Giulio Nittari

**Affiliations:** 1 School of Medicinal and Health Products Sciences, University of Camerino, Camerino, Italy

The authors have done a good job of revision, they have followed all the indications and suggestions provided by the reviewers. The manuscript in the revised version is much more complete, some important concepts have been added and argued. In addition, the manuscript has also taken into consideration the recent scientific literature related to the topic discussed. I believe that in the revised form the manuscript has increased its scientific potential and interest for readers. I consider the revised version of the manuscript suitable for indexing.

Is the study design appropriate for the research question?

Partly

Is the rationale for, and objectives of, the study clearly described?

Partly

Are sufficient details of the methods provided to allow replication by others?

Yes

Are the datasets clearly presented in a useable and accessible format?

Yes

Reviewer Expertise:

Telemedicine, telepharmacy, epidemiology, neurodegeneration disease

I confirm that I have read this submission and believe that I have an appropriate level of expertise to confirm that it is of an acceptable scientific standard.

# Reviewer report: Reviewer response for version 2

**DOI:** 10.5256/f1000research.174047.r338884

**Published:** 2024-11-15

**Authors:** Francesco Buscema

**Affiliations:** 1 University of Turin, Turin, Italy

The authors made substantial updates to the protocol following the suggestions of both reviewers. I have nothing to report except for the lack of a link to the article in reference number 1.

 Jensen HJ, et al., 2019 (Ref 1)

Is the study design appropriate for the research question?

No

Is the rationale for, and objectives of, the study clearly described?

Partly

Are sufficient details of the methods provided to allow replication by others?

Yes

Are the datasets clearly presented in a useable and accessible format?

Not applicable

Reviewer Expertise:

Organizational Health Psychology, Seafarers Health, Psychosocial Risk Evaluation.

I confirm that I have read this submission and believe that I have an appropriate level of expertise to confirm that it is of an acceptable scientific standard.

## References

[1] : Potentially traumatic experiences of seafarers. *J Occup Med Toxicol* .2019;14: 10.1186/s12995-019-0238-9 17 10.1186/s12995-019-0238-9 31164911 PMC6544912

# Reviewer report: Reviewer response for version 1

**DOI:** 10.5256/f1000research.168660.r332925

**Published:** 2024-10-24

**Authors:** Francesco Buscema

**Affiliations:** 1 University of Turin, Turin, Italy

I have carefully reviewed your manuscript. I believe the topic of this systematic review is highly relevant and important for bridging a significant gap in the literature.

 However, to ensure the manuscript meets the highest standards for indexing, I would like to suggest some revisions.

**Protocol Structure:**

 If this manuscript is intended as a protocol, please ensure it adheres to the Guidelines for Preparing a Study Protocol article. The main body of the manuscript should focus on presenting the "Protocol" itself.

**Introduction Section:**

 While the Introduction section generally aligns with the manuscript's objectives, I recommend a comprehensive revision for two primary reasons: 1) Clarity and Grammar and 2) References

 1) Please review the section for grammatical errors and inconsistencies to enhance clarity. 

 2) Ensure that all claims and statements are supported by relevant references.

**Terminology:**

 When discussing individuals working on ships, please use the term "seafarers" rather than "travellers." Seafarers are professionals engaged in maritime work and not simply travelers.

**Eligibility Criteria:**

 To provide a clear understanding of the studies included in your review, please be more specific about the eligibility criteria. The phrase "for example" implies that there are other studies not explicitly listed.

 Please also clarify the specific types of seafarers included in your review. The term "seafarers" can encompass a wide range of workers, such as deck officers, engineers, and catering personnel.

**Exclusion Criteria:**

 Please provide a more detailed explanation of the exclusion criteria. What specific psychosocial well-being outcomes or measures are excluded, and how do you define "organizational factors," "technical aspects," or "non-psychosocial aspects of maritime work"?

**QoL:**

 Please specify the definition of QoL that you will be using in your review.

**Eligibility Criteria for Studies:**

 Consider adding the following sentence to the paragraph related to eligibility criteria: "Studies that provide clear and acceptable definitions for these concepts were considered."

**Definition of QoL:**

 If you intend to use the WHO's definition of QoL, please state this explicitly.

**Other Scales:**

 If you are including scales beyond those explicitly mentioned, please provide a more comprehensive list.

**PRISMA-P Checklist:**

 Please ensure that the PRISMA-P Checklist in the attachment accurately reflects all search strategies conducted, including those using databases other than Scopus.

 I believe that addressing these points will significantly enhance the quality and clarity of your manuscript.

Is the study design appropriate for the research question?

No

Is the rationale for, and objectives of, the study clearly described?

Partly

Are sufficient details of the methods provided to allow replication by others?

Yes

Are the datasets clearly presented in a useable and accessible format?

Not applicable

Reviewer Expertise:

Organizational Health Psychology, Seafarers Health, Psychosocial Risk Evaluation.

I confirm that I have read this submission and believe that I have an appropriate level of expertise to confirm that it is of an acceptable scientific standard, however I have significant reservations, as outlined above.

# Reviewer report: Reviewer response for version 1

**DOI:** 10.5256/f1000research.168660.r332927

**Published:** 2024-10-24

**Authors:** Giulio Nittari

**Affiliations:** 1 School of Medicinal and Health Products Sciences, University of Camerino, Camerino, Italy

Overall a good evaluation of the topic selected.

 some more elaboration and editing is necessary to complete the manuscript.

 In the introduction section, all stressful factors should be mentioned: objective and subjective ones, and then focus to the ones of study. (can use reference: Ref 1

 limitations of study should include except from gender discrepancies, also age discrepancies. Control group should be edited to follow the study group especially in respect of female to male ratio, so as to have a valid conclusion at the end

 Possible treatment/management in order to reduce death anxiety should be expanded also in a paragraph to include frequent psychological consults both on board/telemedicine and off-board, and also a mention of telemedicine to combat the covid pandemic. 

 Some good articles for this section can include:

 1. Ref 2

 2. Ref 3

Is the study design appropriate for the research question?

Partly

Is the rationale for, and objectives of, the study clearly described?

Partly

Are sufficient details of the methods provided to allow replication by others?

Yes

Are the datasets clearly presented in a useable and accessible format?

Yes

Reviewer Expertise:

Telemedicine, telepharmacy, epidemiology, neurodegeneration disease

I confirm that I have read this submission and believe that I have an appropriate level of expertise to confirm that it is of an acceptable scientific standard, however I have significant reservations, as outlined above.

## References

[1] : Factors affecting mental health of seafarers on board merchant ships: a systematic review. *Rev Environ Health* .2024;39(1) : 10.1515/reveh-2021-0070 151-160 10.1515/reveh-2021-0070 36302371

[2] : Evaluation of medical prescriptions and off-label use on board ships to improve healthcare quality. *Eur Rev Med Pharmacol Sci* .2018;22(13) : 10.26355/eurrev_201807_15439 4392-4400 10.26355/eurrev_201807_15439 30024631

[3] : Telemedicine framework to mitigate the impact of the COVID-19 pandemic. *J Taibah Univ Med Sci* .2021;16(2) : 10.1016/j.jtumed.2020.12.010 300-302 10.1016/j.jtumed.2020.12.010 33519334 PMC7833653
